# Refractory ventricular arrhythmia in a patient with suspected immune checkpoint inhibitor-related myocarditis: a case report

**DOI:** 10.1093/ehjcr/ytaf540

**Published:** 2025-10-21

**Authors:** Xinhao Jin, Rui Zheng

**Affiliations:** Department of Critical Care Medicine, Sir Run Run Shaw Hospital, Zhejiang University School of Medicine, Hangzhou, Zhejiang 310016, China; Department of Critical Care Medicine, Sir Run Run Shaw Hospital, Zhejiang University School of Medicine, Hangzhou, Zhejiang 310016, China

**Keywords:** immune checkpoint inhibitors, ICI-induced myocarditis, Myocarditis, Toripalimab, PD-1, Cardio-oncology, Ventricular arrhythmia, Case report

## Abstract

**Background:**

Immune checkpoint inhibitors (ICIs) have revolutionized cancer therapy by modulating immune responses, yet they can trigger rare, life-threatening complications such as myocarditis. Diagnosis remains challenging without confirmatory imaging or biopsy, particularly in haemodynamically unstable patients.

**Case Summary:**

We report a 65-year-old male with oesophageal cancer who developed refractory ventricular tachycardia three months after initiating toripalimab, a PD-1 inhibitor. Clinical suspicion of ICI-related myocarditis was based on ECG abnormalities (polymorphic ventricular tachycardia, ST-segment elevation), elevated troponin I (peak 7450 ng/L), and echocardiography showing reduced left ventricular ejection fraction (30%), with coronary angiography excluding significant obstructive disease. Due to haemodynamic instability, cardiac MRI or biopsy was not feasible. Treatment with high-dose methylprednisolone, immunoglobulin, and anti-arrhythmic drugs (amiodarone, esmolol) yielded limited success, with recurrent arrhythmias requiring multiple cardioversions. Following the family's thoughtful decision to forgo extracorporeal membrane oxygenation, the patient passed away shortly after discharge.

**Discussion:**

This case underscores the diagnostic complexity of suspected ICI-related myocarditis and the critical need for early recognition of immune-mediated cardiac toxicity in immunotherapy patients, emphasizing the role of clinical vigilance when definitive diagnostics are unavailable.

Learning points

- Routine monitoring of ECG and troponin levels is crucial for early detection of ICI-related myocarditis, even in asymptomatic patients.
- Retrograde atrioventricular conduction during ventricular tachycardia is a rare but important electrophysiological finding in ICI-related myocarditis.
- A high index of suspicion and a rapid, integrated approach are essential for diagnosing ICI-related myocarditis when confirmatory tests are unavailable.

## Introduction

Malignant tumours pose a severe global health threat, with 19.3 million new cases and 10 million deaths worldwide in 2020, and projections of 28.4 million cases by 2040.^1^ Immune checkpoint inhibitors (ICIs) have revolutionized cancer treatment by targeting PD-1/PD-L1 and CTLA-4 pathways to enhance anti-tumour immunity.^2,3^ However, ICIs can trigger rare but lethal immune-mediated complications like myocarditis, which is diagnostically challenging due to reliance on clinical findings, Electrocardiograph(ECG), troponin levels, and imaging (confirmatory tests like cardiac MRI or biopsy are often unfeasible in critically ill patients).^4^ Studies indicate myocarditis occurs in 0.09% of patients treated with ICIs,^5^ a rate easily overlooked but significant given widespread use. This case report presents a patient with suspected ICI-related myocarditis complicated by refractory ventricular arrhythmias and retrograde atrioventricular conduction, a rare electrophysiological finding, contributing to the limited literature on this phenomenon.

## Summary figure

**Figure:**
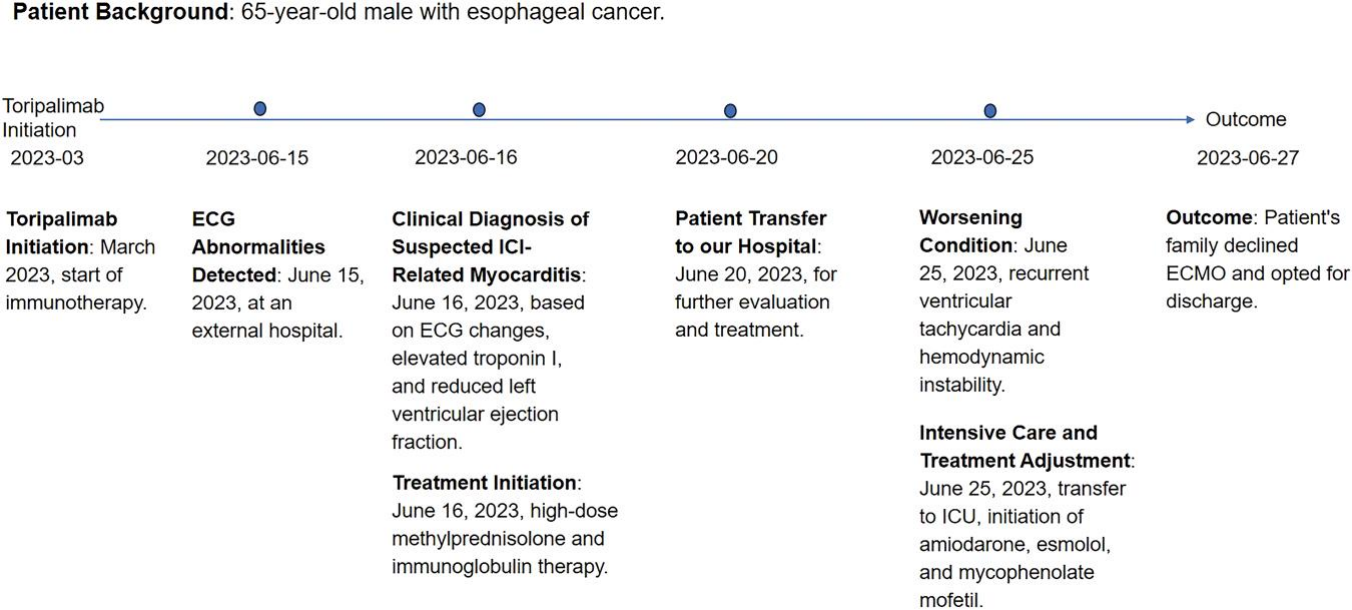


## Case Presentation

**Figure 1:**
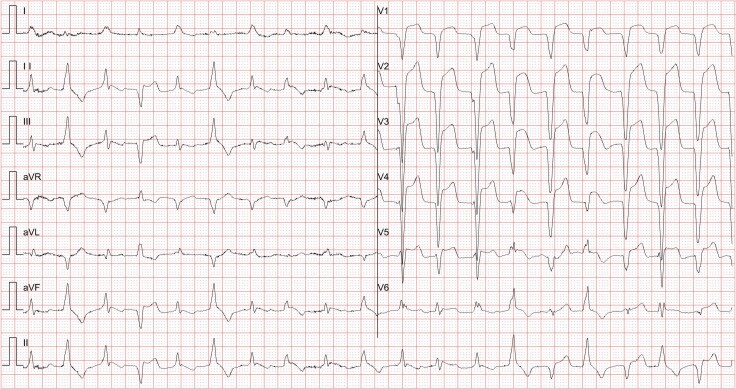
The patient’s pre-admission surveillance ECG showed newly developed abnormalities: atrial fibrillation, complete left bundle-branch block, pathological Q waves over the anteroseptal and anterior walls, and upwardly convex ST-segment elevation, together with frequent multifocal premature ventricular complexes that occasionally coalesced into short runs of ventricular tachycardia.

A 65-year-old male with oesophageal squamous cell carcinoma (cT3N2M0) was admitted on 20 June 2023, with chest tightness and palpitations, 3 months after initiating neoadjuvant therapy (4 cycles of paclitaxel, nedaplatin, and toripalimab, a PD-1 inhibitor.^6^) Routine follow-up five days ago revealed new ECG abnormalities (*Figure 1*): Atrial fibrillation, complete left bundle-branch block with ST-segment elevation, and frequent ventricular ectopy, along with an elevated troponin I of 2.49 μg/L. Echocardiography showed reduced left ventricular function; coronary angiography excluded significant stenosis.

**Figure 2:**
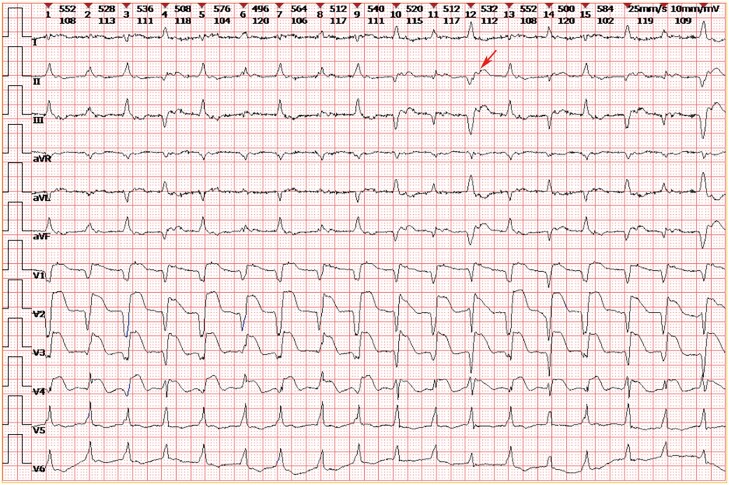
The patient's electrocardiogram (ECG) upon admission indicated: 1.polymorphic ventricular tachycardia. 2.Retrograde atrioventricular conduction(see arrow). 3.Abnormal Q waves with significant ST-segment elevation in the anterior and anterolateral leads. 4.ST-T changes in the inferior and lateral leads.

On admission, laboratory tests showed high-sensitivity troponin I (7450 ng/L, normal 0–54 ng/L), NT-proBNP (16 589 pg/mL), and normal electrolyte levels (potassium 4.48 mmol/L, magnesium 0.99 mmol/L). ECG revealed polymorphic ventricular tachycardia with retrograde atrioventricular conduction, abnormal Q waves with ST-segment elevation in anterior/anterolateral leads, and ST-T changes in inferior/lateral leads(*Figure 2*, marked with arrows indicating retrograde *P* waves). Echocardiography confirmed reduced left ventricular ejection fraction (48.3%), right ventricular enlargement, and moderate tricuspid regurgitation.

**Figure 3:**
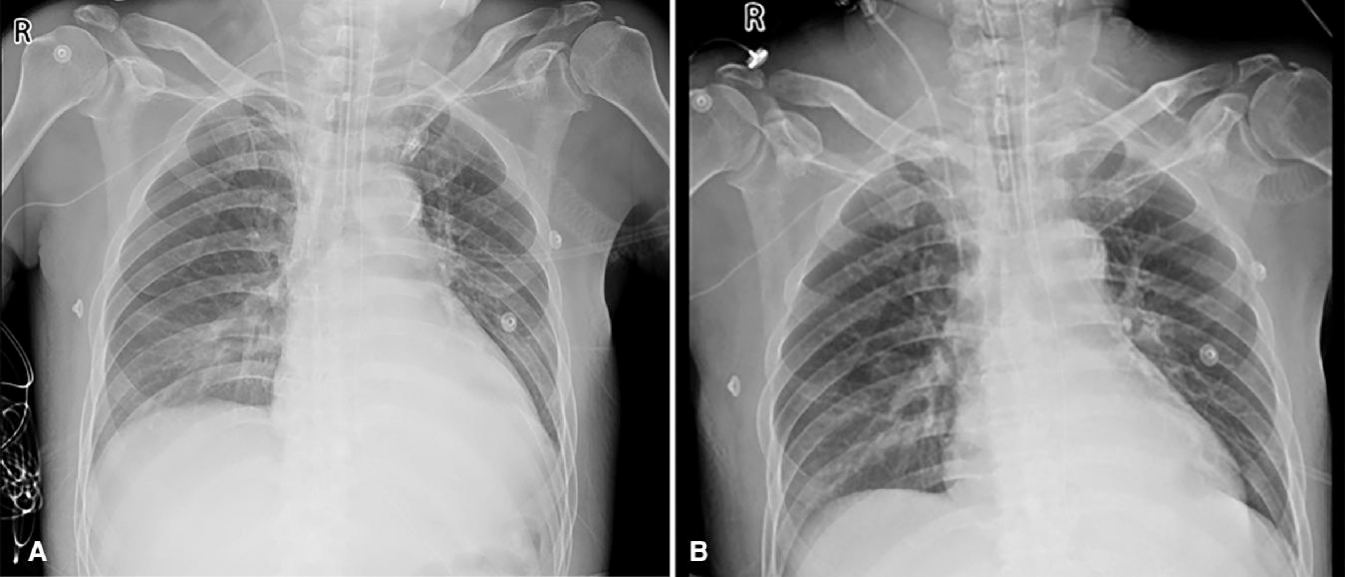
Fig.3A is the chest X-ray of the patient upon admission to the ICU, and Fig.3B is the chest X-ray before the patient's discharge. The images reveal increased lung markings, a small amount of pleural effusion on both sides and patchy blurred shadows in the right lower lung field.

**Figure 4:**
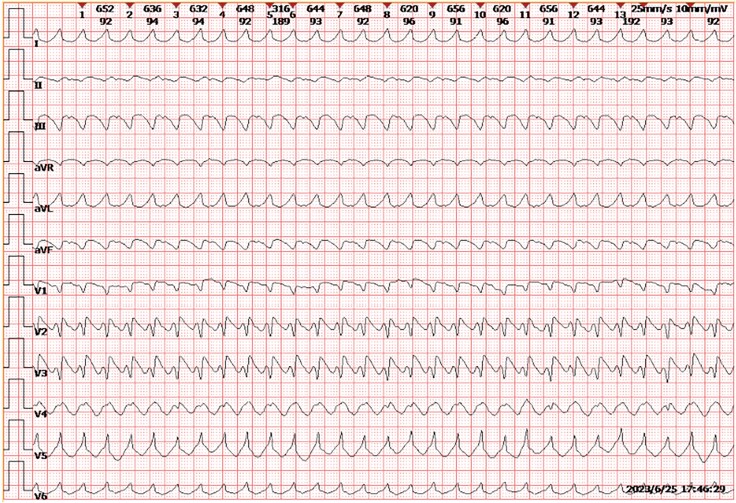
As shown in the figure, the patient frequently experienced ventricular tachycardia during the ICU stay.

**Figure 5:**
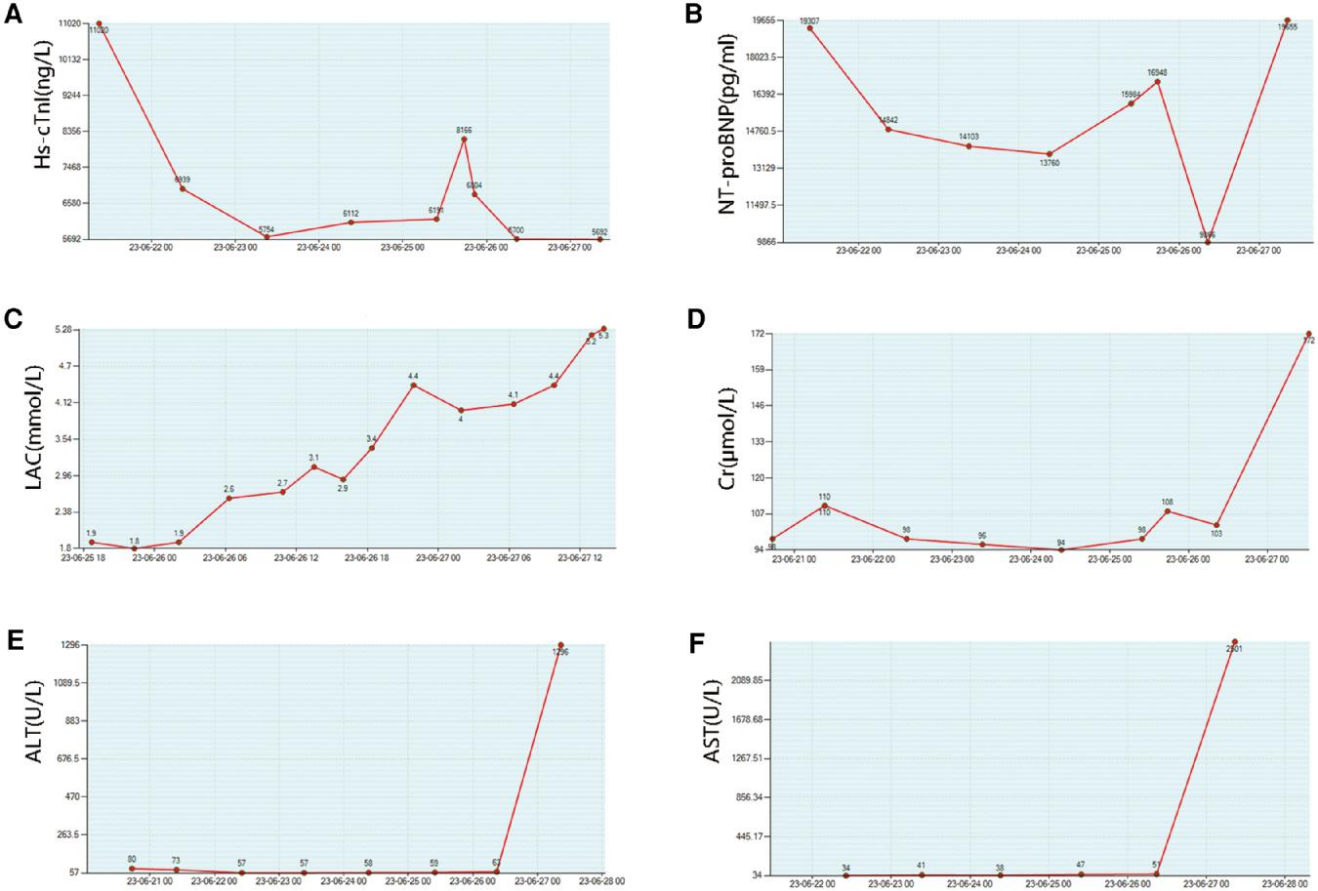
Illustration of the main laboratory markers. (*A*) Time curve of Hs-cTnI during hospitalization. (*B*) Time curve of NT-proBNP during hospitalization. (*C*) Time curve of LAC during hospitalization. (*D*) Time curve of Cr during hospitalization. (*E*) Time curve of ALT during hospitalization. (*F*) Time curve of AST during hospitalization. Hs-cTnI, high-sensitivity cardiac troponin I; NT-proBNP, N-terminal pro-brain natriuretic peptide; LAC, Lactate; Cr, Creatinine; ALT, Alanine aminotransferase; AST, Aspartate Transaminase.

Treatment included high-dose methylprednisolone (240–480 mg/day), intravenous immunoglobulin (10–30 g/day), and amiodarone. Despite this, the patient developed worsening chest tightness and recurrent ventricular tachycardia. Chest X-ray showed increased lung markings, bilateral pleural effusion, and right lower lung patchy shadow (*Figure 3*). He was transferred to the ICU, where frequent ventricular tachycardia with hypotension required repeated cardioversions (*Figure 4*). Therapy was adjusted to esmolol and mycophenolate mofetil, but liver/kidney dysfunction and persistent lactate elevation occurred (*Figure 5*). ECMO was proposed as a potential life-saving measure due to refractory cardiogenic shock, but the family declined, citing concerns about the patient’s quality of life and prognosis, despite discussions highlighting ECMO’s role as a bridge to recovery in severe cases. The patient was discharged at the family’s request and passed away shortly thereafter.

## Discussion

ICI-related myocarditis is a rare but potentially fatal complication, with a median onset of 1–3 months post-ICI initiation,^7,8^ consistent with this case. The uniqueness of this case lies in the documentation of retrograde atrioventricular conduction during ventricular tachycardia (*Figure 2*), a rare electrophysiological finding in ICI-related myocarditis, which has been infrequently reported. Compared to prior reports,^5,7,9^ this case emphasizes the challenge of managing refractory arrhythmias without confirmatory diagnostics and highlights the prognostic implications of family-driven treatment decisions.

Diagnosis was based on clinical criteria (ECG abnormalities, troponin elevation, reduced LVEF) due to the infeasibility of cardiac MRI or biopsy, consistent with guidelines.^7^ Normal electrolyte levels ruled out metabolic causes. The baseline ECG abnormalities detected during routine follow-up underscore the value of proactive monitoring. While routine screening for asymptomatic ICI-treated patients is debated due to low prevalence,^5^ this case suggests that periodic ECG and troponin monitoring may detect subclinical cardiotoxicity, potentially improving outcomes if treated early. Evidence for early intervention is limited but supported by case series showing better outcomes with prompt immunosuppression.^7^

Risk factors for ICI cardiotoxicity remain poorly defined, but combination therapy, pre-existing autoimmune disease, and anti-CTLA-4 use are proposed.^10,11^ Treatment typically involves high-dose glucocorticoids, with immunosuppressants (e.g. mycophenolate mofetil,^12^ alemtuzumab^13^) for steroid-refractory cases. ECMO, considered a bailout therapy for cardiogenic shock, has variable success in ICI-myocarditis.^14,15^ In this case, the family’s decision against ECMO was influenced by the patient’s critical condition and perceived limited benefit, though the patient’s own views could not be elicited due to his unstable state. This highlights the ethical complexity of family-driven decisions in critical care.

## Conclusion

This case underscores the diagnostic and therapeutic complexities of suspected ICI-related myocarditis, particularly when confirmatory tests are unavailable. Clinicians must maintain vigilance for immune-mediated cardiac toxicity in ICI-treated patients, with routine monitoring of troponin, ECG, and cardiac function to detect early abnormalities—even in asymptomatic individuals. Accurate documentation of immunotherapy history is critical for timely diagnosis and management, ultimately improving patient outcomes.

## Data Availability

All data relevant to this case report are included in the manuscript and its figures. No additional datasets were generated or analysed.

## Lead author biography

Dr. Zheng Rui graduated from Wenzhou Medical University. He has a strong interest in critical care medicine, particularly cardiac critical care. Currently, he is working in the field of critical care medicine at Sir Run Run Shaw Hospital.
